# Non-Conventional Features of Plant Oil-Based Acrylic Monomers in Emulsion Polymerization

**DOI:** 10.3390/molecules25132990

**Published:** 2020-06-30

**Authors:** Ananiy Kohut, Stanislav Voronov, Zoriana Demchuk, Vasylyna Kirianchuk, Kyle Kingsley, Oleg Shevchuk, Sylvain Caillol, Andriy Voronov

**Affiliations:** 1 Department of Organic Chemistry, Institute of Chemistry and Chemical Technologies, Lviv Polytechnic National University, 79000 Lviv, Ukraine; ananiy.kohut@gmail.com (A.K.); stanislav.voronov@gmail.com (S.V.); vasuluna411@ukr.net (V.K.); oshev2509@gmail.com (O.S.); 2 Department of Coatings and Polymeric Materials, North Dakota State University, Fargo, ND 58108-6050, USA; zoriana.demchuk@ndsu.edu (Z.D.); kyle.kingsley.2@ndsu.edu (K.K.); 3 ICGM, Univ Montpellier, CNRS, ENSCM, 34095 Montpellier, France; sylvain.caillol@enscm.fr

**Keywords:** biobased polymers, plant oil-based monomers, mixed micelles, methyl-β-cyclodextrin inclusion complex, emulsion polymerization

## Abstract

In recent years, polymer chemistry has experienced an intensive development of a new field regarding the synthesis of aliphatic and aromatic biobased monomers obtained from renewable plant sources. A one-step process for the synthesis of new vinyl monomers by the reaction of direct transesterification of plant oil triglycerides with *N*-(hydroxyethyl)acrylamide has been recently invented to yield plant oil-based monomers (POBMs). The features of the POBM chemical structure, containing both a polar (hydrophilic) fragment capable of electrostatic interactions, and hydrophobic acyl fatty acid moieties (C_15_-C_17_) capable of van der Waals interactions, ensures the participation of the POBMs fragments of polymers in intermolecular interactions before and during polymerization. The use of the POBMs with different unsaturations in copolymerization reactions with conventional vinyl monomers allows for obtaining copolymers with enhanced hydrophobicity, provides a mechanism of internal plasticization and control of crosslinking degree. Synthesized latexes and latex polymers are promising candidates for the formation of hydrophobic polymer coatings with controlled physical and mechanical properties through the targeted control of the content of different POBM units with different degrees of unsaturation in the latex polymers.

## 1. Introduction

The problem of depletion of fossil raw materials has become of global importance and is complicated not only by economic but also environmental and political factors [1]. However, synthetic polymers are still one of the most widely used materials in everyday use. Environmental analysis shows that huge amounts of plastic waste are found in the environment, and their contribution to the ever-increasing amount of solid waste is a significant environmental threat [2]. Plant oils are low-cost raw materials for the manufacture of monomers and polymers [3,4,5], which have several advantages over conventional polymeric materials, namely biodegradability, non-toxicity, biocompatibility, and hydrophobicity. Therefore, an important task for synthetic chemists is to consider renewable raw materials as an alternative to raw counterparts of fossil origin. Due to the wide range of plant oils, the variety of their chemical compositions, and, in many cases, abandon of resources, they became an interesting object to be used in polymers synthesis as a renewable raw material.

The total production of plant oils in the world in 2019 amounted to more than 200 million tons, of which about 15% are used as raw materials for the synthesis of new chemical compounds and materials [6]. The presence of plant oils or their derivatives (fatty acids) in various compositions of polymeric materials improves their optical (gloss), physical (flexibility, adhesion) [7,8], and chemical (resistance to water and chemicals) properties [9]. The synthesis of biobased monomers from renewable resources is a promising platform for the synthesis and implementation of new environmentally friendly industrial polymer materials [10]. However, biomass molecules rarely possess suitable reactive functions for radical polymerization. Indeed, the double bonds of vegetable oils are poorly reactive in radical polymerization. Therefore, synthesizing radically polymerizable biobased monomers is a real challenge [11]. It should be noted that methods for the synthesis of vinyl monomers based on soybean, olive, and linseed oils through plant oil direct transesterification with *N*-(hydroxyethyl)acrylamide [10,12,13] has been recently developed along with methods of their copolymerization [14]. The ability of these plant oil-based monomers (POBMs) to undergo free radical (co)polymerization reactions has been confirmed and demonstrated [12,14,15]. The conversion, polymerization rate, and molecular weight of the polymers have been shown to depend on unsaturation of the plant oil chosen for POBMs synthesis [12,14].

## 2. Features of Synthesis of Vinyl Monomers from Plant Oils

**Figure 1:**
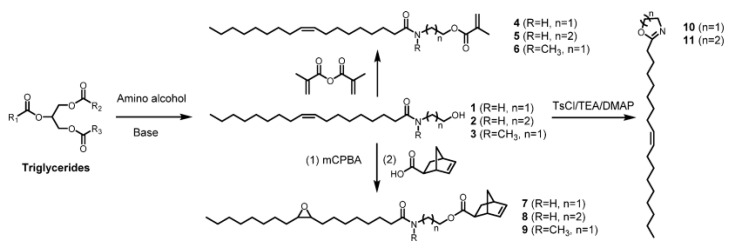
Transformation of triglycerides in N-hydroxyalkylamides and methacrylate monomers. Reprinted with permission from [16]. Copyright (2015) American Chemical Society.

Tang et al. [16] reported on the synthesis of *N*-hydroxyalkylamides and methacrylate hydrophobic monomers through the interaction of plant oil triglycerides with amino alcohols (Figure 1). They developed a new interesting approach that involves the use of a two-step process. In the first stage, the interaction of triglycerides with amino alcohols with the formation of *N*-hydroxyalkylamides by the amidation reaction:

Aminoalcohols, namely ethanolamine (R = H, n = 1), 3-amino-1-propanol (R = H, n = 2), and N-methylethanolamine (R = CH_3_, n = 1), were used as chemicals which enables the synthesis of a wide range of new monomers. In the second stage, the corresponding monomers were synthesized by the interaction between N-hydroxyethylamides R’–C(O)–N(R)–(CH_2_)_n_–CH_2_OH and methacrylic anhydride. In such a way, methacrylate monomer CH_2_=C(CH_3_)–C(O)–O–CH_2_–(CH_2_)_n_–N(R)–C(O)– (CH_2_)_7_–CH=CH–(CH_2_)_7_–CH_3_ based on high oleic soybean oil was synthesized.

A study of free radical polymerization of these monomers revealed that the double bonds in the fatty acid chains remain unaffected during the reaction. The synthesized methacrylate monomers based on high oleic soybean oil, depending on the nature of the branches in the side chain, have a wide range of glass transition temperatures [16]. In further studies, Tang and co-authors used more than twenty amino alcohols to establish the mechanism of amidation of plant oils, in particular high-oleic soybean oil [8]. Depending on the nature of the acyl fatty acid and amide structures, the polymers have a wide range of glass transition temperatures – from viscoelastic to thermoplastic materials. The possibility of controlled hydrogenation of unsaturated double bonds was also shown, which provides control over thermal and mechanical properties of polymers [8].

On the other hand, Harrison and Wheeler have shown for the first time [17] that the polymerization rate decreases with increasing unsaturation degree of acrylates containing acyl moieties of unsaturated fatty acids. They explained that the retarding of the polymerization rate and the low conversion are due to the interaction of radicals with the mobile hydrogen atoms in the acyl moieties. This leads to the formation of new allylic radicals of low activity which retards propagation of polymer chains (chain transfer reaction, retardation of the process) [17].

Through the esterification reaction of fatty alcohols with acryloyl chloride, Chen and Bufkin synthesized a range of acrylates, including linoleyl acrylate, oleyl acrylate, and lauryl acrylate, and studied their copolymerizability. They showed that the presence of fatty fragments in acrylate molecules determines their polarity, which leads to alternating copolymerization with methyl methacrylate [18].

In their next study [19], Chen and Bufkin confirmed Harrison’s research results [17] and showed that the presence of fatty acid moieties in the structure of the new acrylates leads to a decrease in both the polymerization rate and polymer molecular weights because of chain transfer reactions. Increasing the content of unsaturated acyl groups in the copolymer also reduces its glass transition temperature. The authors suggested the mechanisms for crosslinking acrylate copolymers and studied their physical and mechanical properties [19].

**Figure 2:**
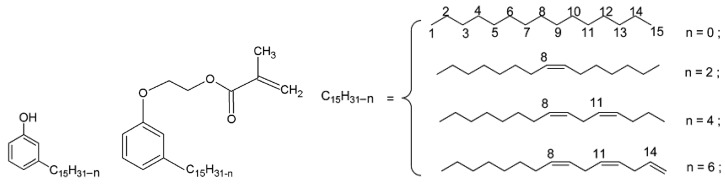
Structure of cardanol and a cardanol-based monomer. Adapted from [22] with permission from The Royal Society of Chemistry.

Through amidation of triglycerides with amino alcohols, natural derivatives of surfactants and lubricants were obtained [20]. It was demonstrated that the high reactivity of the vinyl group of the acrylate monomers based on plant oils determines the course of their polymerization and copolymerization by a free radical mechanism [21]. Caillol et al. [22,23] proposed methods for the synthesis of a new monomer from cardanol (Figure 2), which is isolated from cashew nuts. Reactivity of the cardanol-based monomer in the copolymerization reactions with the POBMs was studied [24].

Cardanol-based polymers have a controlled flexibility due to “internal plasticization”, which occurs due to the presence of a long side chain. The presence of these fragments also provides polymeric materials based on cardanol with hydrophobic properties. It should be noted that cardanol exhibits antimicrobial and anti-thermite properties and, under certain conditions, can provide them to copolymers based on it. It is compatible with a wide range of polymers, such as alkyds, melamines, polyesters, etc. [22].

In the study [23], synthetic pathways to molecular blocks from cardanol by one- or two-stage methods were reported. In particular: 1) dimerization/oligomerization of cardanol, to increase its functionality; 2) synthesis of reactive amines by thiolene coupling; 3) epoxidation and (meth)acrylation of cardanol for the synthesis of polymers and materials with oxirane or (meth)acrylate groups.

A feature of cardanol-based monomers and polymers is the presence of aromatic fragments and fatty moieties in their structure at the same time. The authors showed that cardanol-based polymeric materials have prospective properties for manufacturing coatings, in particular, with enhanced mechanical and thermal properties [23].

**Figure 3:**
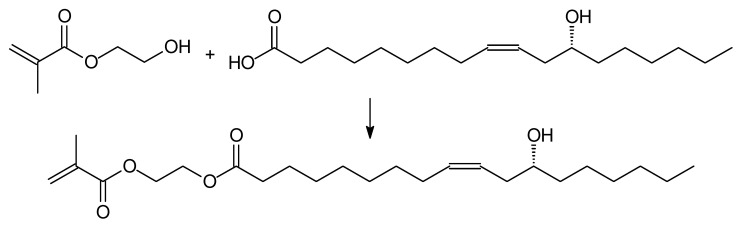
Schematic of synthesis of methacrylated ricinoleic acid monomer.

Recently, methacrylated ricinoleic acid monomer (Figure 3) was synthesized by the esterification reaction of ricinoleic acid with 2-hydroxyethyl methacrylate [25]:

Other plant oil-based methacrylates have been widely studied in atom transfer radical polymerization (ATRP). A method for the ATRP of lauryl methacrylate was developed by Mandal et al. to obtain narrowly distributed polymers with predefined molecular weights [26]. Fatty alcohol derived methacrylates were used to prepare well defined main chain substituted polymethacrylates by copper-mediated ATRP [27].

It should be noted that all known methods for the synthesis of monomers from plant oil triglycerides are multi-stage and involve several steps, which makes their implementation in industry more challenging.

**Figure 4:**
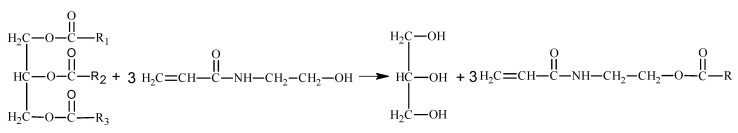
Schematic of synthesis of acrylic monomers based on plant oil triglycerides where R_1_, R_2_, R_3_ are saturated and unsaturated fatty acid chains with one or several double bonds. Reprinted with permission from [10]. Copyright (2015) American Chemical Society.

Recently, we developed a one-step process for the synthesis of new vinyl monomers by the direct transesterification reaction of plant oil triglycerides with *N*-(hydroxyethyl)acrylamide (Figure 4) [13,28].

Using this process, a range of POBMs was synthesized [10,12]. In the reaction, *N*-(hydroxyethyl)acrylamide can be considered as an alcohol ROH – where the unsaturated fragment CH_2_=CH–C(O)–NH–CH_2_–CH_2_– is a residue –R. Upon the alcoholysis (transesterification), a residue exchange between the triglyceride and *N*-(hydroxyethyl)acrylamide occurs leading to formation of the corresponding monomers.

The POBMs synthesis was conducted in THF with an excess of *N*-(hydroxyethyl)acrylamide (molar ratio of *N*-(hydroxyethyl)acrylamide to triglyceride as 5.9: 1) in order to achieve complete transesterification of the triglycerides. The yield of the desired monomers was about 94–96%. The reaction by-product (glycerol) and the excessive *N*-(hydroxyethyl)acrylamide are easily removed by washing with brine after diluting the reaction mixtures with CH_2_Cl_2_. The POBMs sparingly soluble in water remain in the organic phase. To avoid free radical polymerization, 2,6-di-*tert*-butyl-p-cresol (0.05 to 0.1% by weight) was added as an inhibitor to the reaction mixtures prior the synthesis.

Upon the alcoholysis of olive oil with *N*-(hydroxyethyl)acrylamide, 2-*N*-acryloylaminoethyl oleate CH_2_=CH–C(O)–NH–CH_2_CH_2_–O–C(O)–(CH_2_)_7_–CH=CH–(CH_2_)_7_–CH_3_ is predominantly formed.

This monomer contains one double bond in the acryloylamide moiety and one double bond in the fatty acid chain. Such a structure, in comparison with the soybean oil-based monomer 2-*N*-acryloylaminoethyl linoleate (CH_2_=CH–C(O)–NH–CH_2_CH_2_–O–C(O)–(CH_2_)_7_–CH=CH–CH_2_– CH=CH–CH_2_–(CH_2_)_3_–CH_3_) reduces the extent of the chain transfer reaction. Accordingly, fewer less active radicals are formed and the polymerization reaction proceeds to higher conversion with a less pronounced retardation effect. This leads to the formation of polymers (copolymers) with a higher molecular weight and a lower polydispersity index.

**Figure 5:**
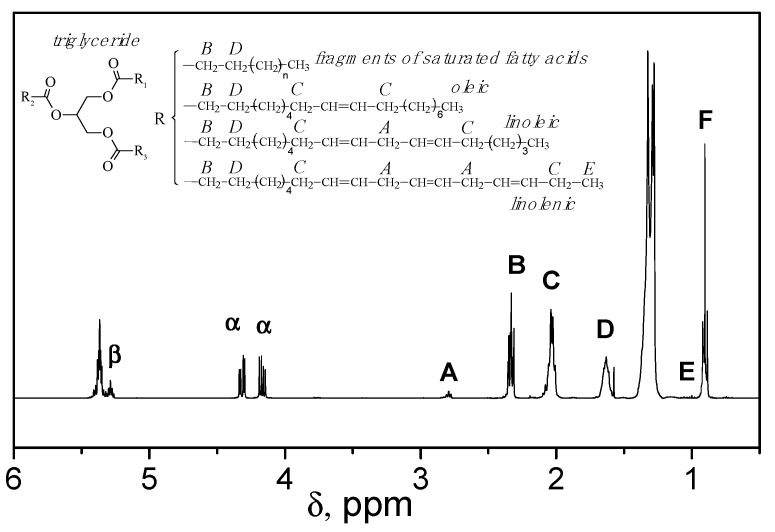
^1^H-NMR spectrum of olive oil. Adapted with permission from [12]. Copyright (2016) American Chemical Society.

The composition of plant oils is known to depend on the type of crop raw material. Therefore, an important step was to determine the content of different fatty acid chains in the mixture of monomers obtained upon the transesterification of olive oil with *N*-(hydroxyethyl)acrylamide. The content of fatty acid chains in the oil was determined from the integral intensities of the characteristic proton signals of each fatty acid chain and glycerol fragments using ^1^H-NMR spectroscopy of the plant oil [29] (Figure 5).

The content of the linolenic acid units (linolenate) in olive oil was calculated by measuring the integral value of the signal at 1 ppm, which corresponds to the protons of the methyl group in a linolenic acid chain (signal E in Figure 5). Taking into account only one of the signals of α-protons of glycerol, i.e., the signal at 4.27 ppm (Figure 5), the ratio of the integral values is two α-glycerol protons to three protons of the linolenic acid methyl group. It should be kept in mind that three linolenic acid chains can undergo transesterification in the same glycerol molecule. A field correction factor is the ratio of two protons of glycerol to nine protons of the linolenic acid methyl groups. By converting the ratio of these areas to percentages, a ratio of 22.2% glycerol to 100% linolenic acid was obtained [29]. Calibration of the integral value of one of the glycerol α-proton signals to the value in the ^1^H-NMR spectrum in the signal region at 0.98 ppm directly gives the percentage of linolenic acid in the sample −0.73%.

The percentage of linoleic acid chains (linoleates) in olive oil was determined from the ratio of the integral value of the signal at ~2.74 ppm, which corresponds to the methylene protons between two double bonds (signal A in Figure 5), to the integral value of one of glycerol α-protons. The field correction factor (33.3) is the ratio of two glycerol protons to six possible methylene protons in linoleate. The amount of linoleic acid is calculated by subtracting the twiced content of linolenic acid, determined from the peak at 2.74 ppm [26].

The percentage of oleic acid chains (oleates) was determined from the ratio of the integral value of the signal at ~2.02 ppm, which corresponds to the protons in the α-position to the double bond of all unsaturated fatty acids (signal C in Figure 5), to the integral value of one of glycerol α-protons. Accordingly, the ratio of two glycerol protons to twelve possible protons in the α-position to the double bond of all unsaturated fatty acids is the field correction factor equal to 16.7. The percentage of oleic acid was calculated by subtracting the content of unsaturated linolenic and linoleic acid chains from the determined value [29].

The content of saturated fatty acid chains was determined from the fact that the total content of fatty acids is 100%, and the amount of unsaturated fatty acid chains was subtracted from 100%. The determined content of fatty acid chains is given in Table 1. These data are in a good agreement with the literature data obtained by gas chromatography of olive oil [30].

Thus, according to ^1^H-NMR spectroscopy, the olive oil triglycerides include: saturated fatty acid chains (C18:0) −12.44%; oleic acid chains (C18:1) −80.23%; linoleic acid chains (C18:2) −6.60%; and linolenic acid chains (C18:3) −0.73%.

These results are consistent with the literature data obtained by gas chromatography of olive oil, where the saturated chains make up 1–20%, oleic acid chains 55–83%, linoleic acid chains 3.5–21%, and linolenic acid chains less than 1% [31]. Using this approach, monomers based on various plant oils were successfully synthesized and characterized (Table 2).

**Figure 6:**
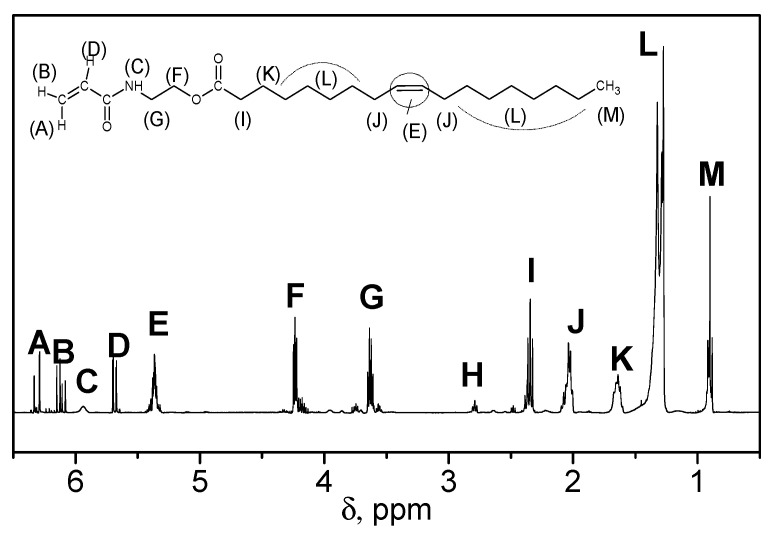
^1^H-NMR spectrum of the olive oil-based monomer. Adapted with permission from [12]. Copyright (2016) American Chemical Society.

The monomer structure was confirmed by FTIR and ^1^H-NMR spectroscopy [12]. A ^1^H-NMR spectrum of the olive oil-based monomer is shown in Figure 6. Similar spectra are recorded for other POBMs, which confirms the presence of the acryloylamide moiety in their molecules. This allows for predicting similar reactivity of the vinyl group in the monomers acryloylamide moiety in the radical polymerization [12].

**Figure 7:**
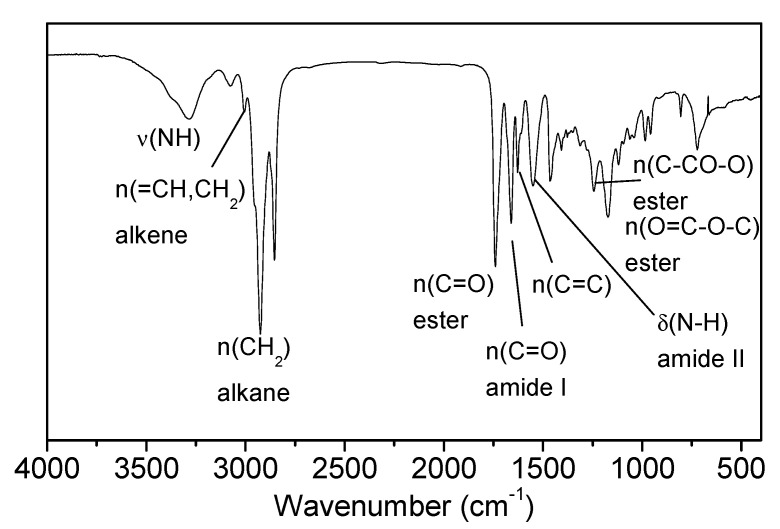
FTIR spectrum of the olive oil-based monomer. Adapted with permission from [12]. Copyright (2016) American Chemical Society.

The FTIR spectroscopy data indicate the addition of fatty acid acyl moieties to the acrylamide fragment (e.g., for the olive monomer—Figure 7). FTIR spectra of monomers based on other plant oils are pretty similar. Hence, the synthesized POBMs can be attributed to the conventional vinyl monomers because the acryloylamide moiety provides the participation of these monomers in the free radical polymerization.

**Figure 8:**
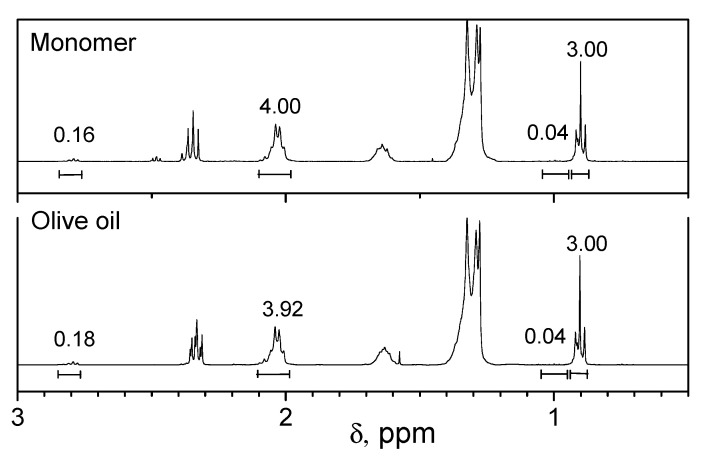
^1^H NMR spectra of the olive oil and the olive oil-based monomer.

The content of fatty acid chains in the monomer mixture produced by the transesterification of plant oils with *N*-(hydroxyethyl)acrylamide was determined on the example of the olive monomer by calculating the ratio of the integral values in the ^1^H-NMR spectra of the commercial oil and the resulting monomer mixture (Figure 8).

According to the ^1^H-NMR spectroscopy data, the monomer mixture consists of: saturated fatty acid chains (C18:0) −11.47% (stearic and palmitic); oleic acid chains (C18:1) −81.9%; linoleic acid chains (C18:2) −5.9%; and linolenic acid chains (C18:3) −0.73% after the olive oil transesterification.

Thus, the major component of the olive monomer is a monomer with oleic acid chains (81.9%) – 2-*N*-acryloylaminoethyl oleate. The soybean monomer is mainly a monomer with linoleic acid chains (57.5%) – 2-*N*-acryloylaminoethyl linoleate whereas the linseed monomer predominantly consists of a monomer with linolenic acid chains (58%) – 2-*N*-acryloylaminoethyl linolenate.

One of the most important characteristics of monomers is the degree of unsaturation, which for POBMs is determined by the number of the double bonds in the fatty acid chains. To compare the monomers in terms of the unsaturation degree, their iodine values were determined and compared with those for the corresponding plant oils used for the synthesis of the POBMs (Table 2). The obtained results show that the iodine value for the monomers is higher than those for the oils, due to the presence of an unsaturated acryloylamide moiety. Depending on the type of oil, the iodine values for various monomers differ. For instance, the iodine value for 2-*N*-acryloylaminoethyl oleate (110 g/100 g) is significantly lower than that for 2-*N*-acryloylaminoethyl linoleate (149 g/100 g) due to the different unsaturation degree of the molecules. Besides the acryloylamide moiety, there are two double bonds in the fatty acid chain of the soybean monomer molecule [10]. The low solubility of these monomers in water implies their highly hydrophobic nature [12].

**Figure 9:**
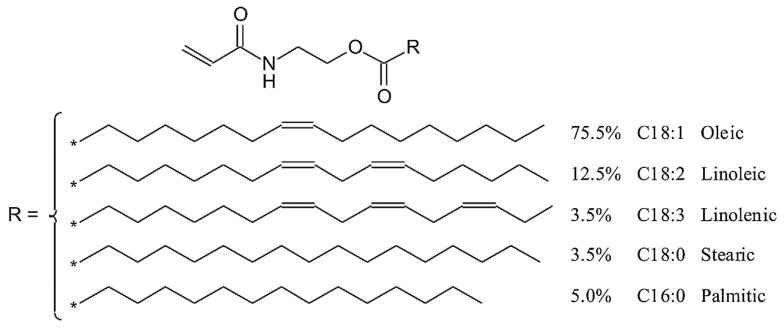
General formula of the plant oil-based monomers. Reprinted from [32]. Copyright (2018), with permission from Elsevier.

Analysis of the data obtained from the synthesis and characterization of plant oil-based acrylic monomers allows for drawing the POBM general formula (Figure 9) as:

## 3. Features of Homo- and Copolymerization of Plant Oil-Based Acrylic Monomers

The reactivity of the POBMs in the free radical polymerization reactions (chain growth reaction) is determined by the presence of an acryloylamide moiety containing a vinyl group which is common for all monomers. However, the POBMs contain a certain amount of unsaturated fatty acid chains of various structures (the different numbers of both double bonds and hydrogen atoms in the α-position to the double bonds). This causes the monomers to participate in chain transfer reactions due to the abstraction of the allylic hydrogen atoms and the formation of less active radicals. Hence, the monomer chain transfer constants C_M_ clearly depend on monomer structure as follows: 0.033 (most unsaturated LSM) > 0.026 (SBM) > 0.023 (SFM) > 0.015 (least unsaturated OVM) with respect to decreasing number of C−H groups in the α-position of the fatty acid double bonds) [12]. This impacts the polymerization conversion and molecular weights of the resulting polymers (copolymers) from the POBMs.

The features of homopolymerization kinetics for 2-*N*-acryloylaminoethyl oleate revealed that the orders of reaction with respect to monomer and initiator are 1.06 and 1.2, respectively. The unsaturation degree of fatty acid chains in the POBM molecules was used as a criterion for comparing the kinetic data of homopolymerization of 2-*N*-acryloylaminoethyl oleate and 2-*N*-acryloylaminoethyl linoleate. The observed deviations of the orders of reaction are due to the specific mechanism of the homopolymerization reaction of 2-*N*-acryloylaminoethyl oleate which includes two simultaneous reactions (chain propagation and transfer) [12]. Although the propagating radicals might be very reactive, once the chain is transferred to the allylic C−H, the newly formed radical becomes more stable due to resonance stabilization and does not readily initiate new chains. In comparison with the soybean monomer, the olive monomer is less involved into the chain transfer reactions (the chain transfer constant C_M_ = 0.015 for OVM while for SBM C_M_ = 0.026). As a result, the homopolymerization of the olive monomer occurs at a higher rate of 12.2–45.3 · 10^−5^ mol/(L·s) when compared with the more unsaturated soybean monomer −4.3−11.3·10^−5^ mol/(L·s). The molecular weights of the homopolymers were determined by gel permeation chromatography. The resulting homopolymers from OVM have higher number average molecular weights (16 800–23 200 g/mol for 2-*N*-acryloylaminoethyl oleate compared to 13 600–14 300 g/mol for 2-*N*-acryloylaminoethyl linoleate).

The reactivity of the POBMs in chain copolymerization was studied in their reactions with styrene and vinyl acetate. A characteristic feature of the POBMs is the presence of the acryloylamide fragment (CH_2_=CH–C(O)–NH–CH_2_–CH_2_–) in their structure, which determines the monomer reactivity in copolymerization. The composition of the copolymers was determined from ^1^H-NMR spectroscopy data. Radical copolymerization of the plant oil-based monomers is described with the classical Mayo–Lewis equation. The Alfrey−Price Q (0.41–0.51) and e (0.09–0.28) parameters are close for all POBMs and do not essentially depend on the monomer structure [13]. This is due to the presence of the same acryloylamide moiety in the POBM molecules which determines the monomer reactivity in polymerization reactions.

The features of copolymerization are determined by the structure of monomers based on plant oil triglycerides along with the degradative chain transfer by formation of less active radicals. Nevertheless, the growth of macrochains can be described with the conventional features of chain copolymerization, yielding copolymers with a potential to be used in a broad variety of applications.

Vinyl monomers from plant oils that have different degrees of unsaturation, soybean, and olive oils, were copolymerized in emulsion with styrene to investigate the kinetics features and feasibility of latex formation. The kinetics of emulsion copolymerization of styrene with the olive and soybean monomers agree with the Smith-Ewart theory since the number of nucleated latex particles is proportional to the surfactant and initiator concentration to the powers 0.58–0.64 and 0.39–0.46, respectively [33]. Copolymerization of styrene with POBMs follows the typical phenomenology for emulsion polymerization of hydrophobic monomers with a micellar nucleation mechanism. When the POBMs were copolymerized in emulsion with significantly more water soluble comonomers, methyl methacrylate and vinyl acetate, latex particle nucleation mainly occurred through homogeneous mode. For the emulsion copolymerization of methyl methacrylate with OVM and SBM, the reaction orders with respect to emulsifier and initiator are 0.33–0.67 and 0.56–0.69, respectively. It was shown that upon adding the highly hydrophobic POBMs into the monomer mixture, the latex polymer particles originating from micellar nucleation essentially increases [15].

Using emulsion and miniemulsion copolymerization of the olive and soybean monomers with styrene or methyl methacrylate, stable aqueous dispersions of polymers with latex particle sizes of 40–210 nm were produced. The content of the POBM units in the macromolecules of the latex polymers is 5–60 wt.%. The average molecular weight of the synthesized polymers varies in the range of 30,000–391,500. It was found that the molecular weight of the latex copolymers decreases with increasing unsaturation degree of the POBMs and their content in the reaction mixture, which is explained by degradative chain transfer to unsaturated fatty acid chains [15,33].

An analysis of the literature shows a rapid development of a new branch in polymer science related to the chemistry of aliphatic and aromatic biobased monomers obtained from renewable plant sources. The synthesis of such monomers allows for producing fully biobased polymers with biocompatible and biodegradable properties which do not pollute the environment. Copolymerization of monomers synthesized from various plant oils, which have different unsaturation, enables formation of copolymers with side branches of the macrochain with different unsaturation degrees. A large variety of the POBMs allows to synthesize polymers having moieties with different unsaturation and to make coatings with adjustable cross-linking degree thereof, including latex copolymers from fully biobased monomer mixtures [24]. Remarkably, copolymerization of the POBMs with commercial vinyl monomers enables synthesis of polymers with enhanced hydrophobicity along with the mechanism of internal plasticization.

Synthesized latex polymers and copolymers are prospective candidates for the formation of moisture-/water-resistant polymer coatings with controlled physical and mechanical properties using controlled content of incorporated POBM units with different unsaturation in the latex structure.

## 4. Preparation of Polymeric Coatings from Plant Oil-Based Monomers

As shown by Solomon [34], both crude and modified plant oils are common film-forming materials for the production of paints and varnishes. Despite the emergence of various synthetic polymers based on vinyl and acrylic monomers, crude plant oils are still the basis of some paints for painting the roofs of houses and other outdoor objects. One of the disadvantages of such materials is the slow drying and relatively low moisture resistance. The synthesis of plant oil-based acrylic monomers opens new avenues for producing homo- and copolymers thereof with adjustable physical and mechanical properties (e.g., flexibility, strength, *etc.*) due to formation of three-dimensional networks with controlled cross-linking density. The development of such coatings was shown to be carried out through oxidative cross-linking of the polymer network [35].

It should be noted that upon POBM application for the synthesis of latex polymers as highly hydrophobic film-forming protective coatings, they form three-dimensional networks with adjustable cross-linking density through the oxidative cross-linking mechanism.

**Figure 10:**
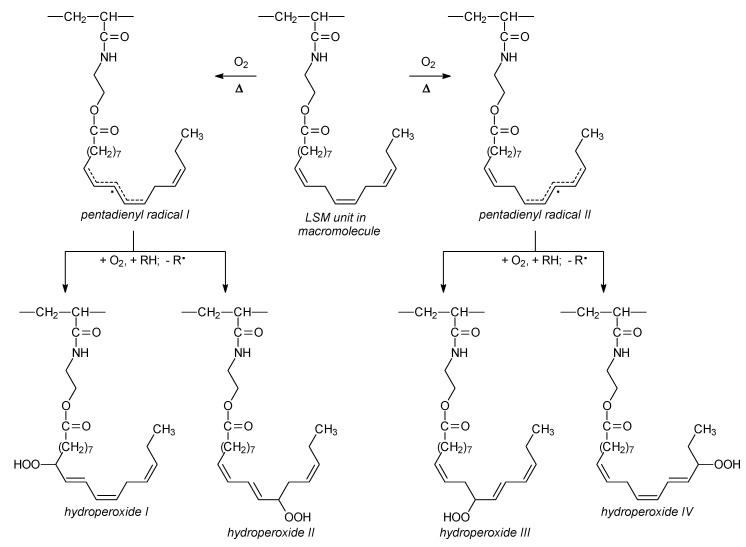
Formation of radicals and hydroperoxides during oxidative cross-linking of polymers from linseed oil-based monomer (LSM).

Triglycerides of linseed oil contain about 52% of linolenic (*cis*,*cis*,*cis*-9,12,15-octadecatrienoic) acid chains which have three isolated carbon-carbon double bonds in their structure (Figure 10). Other fatty acids in linseed oil are oleic (*cis*-9-octadecenoic, 22%) and linoleic (*cis*,*cis*-9,12-octadecadienoic, 16%) acids. Hence, linolenate is a major constituent of LSM.

Oxidative cross-linking of the LSM-based copolymers is a free radical chain process consisting of chain initiation, propagation, and termination steps. Initiation, i.e., the formation of a fatty acid chain radical, can occur by thermal homolytic cleavage of a C–H bond or by a hydrogen atom abstraction from C–H by an initiator free radical. The bond dissociation energy of a bisallylic hydrogen is approximately 42 kJ/mol lower than that of an allylic hydrogen. As a result, linoleates and linolenates are more readily autooxidized and cross-linked in comparison with oleates.

**Figure 11:**
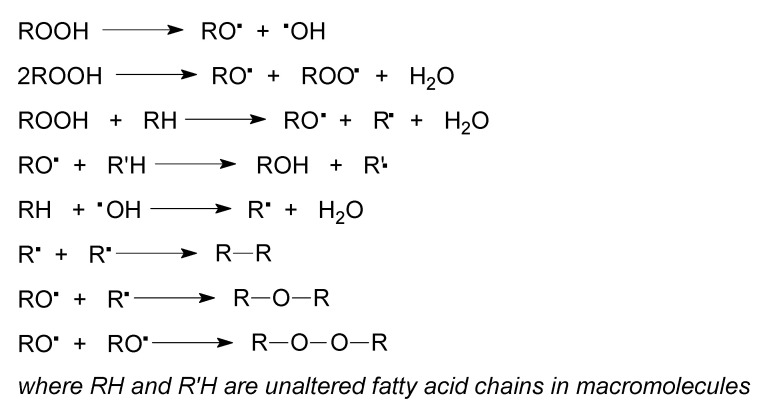
Oxidative cross-linking of polymers from linseed oil-based monomer (LSM).

Hydrogen atom abstraction from C-11 or C-13 of the linolenate moiety leads to pentadienyl radicals I and II (Figure 10). Subsequent oxygen addition to radicals I and II generates peroxyl radicals which are able of abstracting a hydrogen atom from a donor such as another linolenate moiety to form conjugated *trans,cis*-hydroperoxides I–IV (Figure 10). The hydroperoxides undergo decomposition reaction (Figure 11). The last three reactions lead to cross-linking of the macromolecules and formation of a polymer network.

The main mechanical characteristics of films from latex polymers based on styrene or methyl methacrylate and the POBMs were determined. The composition of the copolymers was calculated from the ^1^H-NMR spectroscopy data. The content of the olive and soybean monomers in the reaction mixture varied from 10 to 40 wt. % for copolymers with methyl methacrylate and from 25 to 60 wt. % for copolymers with styrene. A plasticizing effect and enhanced hydrophobicity were observed for the copolymers synthesized from 2-*N*-acryloylaminoethyl oleate and 2-*N*-acryloylaminoethyl linoleate as comonomers. The unsaturation degree of the olive and soybean monomers was used as an experimental parameter to control the latex properties. The effect of monomer unsaturation on the cross-linking density of the latex films and, thus, on the physical and mechanical properties of the coatings was demonstrated [35].

The decrease in the glass transition temperature of the latex copolymers (from 105 to 57 °C for the copolymers of the olive and soybean monomers with methyl methacrylate and from 100 to 5 °C for the copolymers with styrene) indicates that the presence of the OVM and SBM units in the macromolecules affects thermomechanical properties of the resulting latex copolymers. The olive and soybean monomer units impart flexibility to the macromolecules, improve the conditions of film formation, increase the strength in comparison with conventional polystyrene and poly(methyl methacrylate). Moreover, the OVM and SBM units in the macromolecules increase the hydrophobicity of polymeric latex films thus reducing the negative impact of water on the properties of the coatings.

Therefore, the incorporation of the hydrophobic fatty acid chains into the macromolecules of latex polymers allows for formation of polymer networks with a controlled cross-linking density along with enhancing the water resistance of the coatings. The copolymers based on the olive and soybean monomers enable the formation of coatings with low surface energy and water-repellent properties.

## 5. Formation of Micelles from the Complexes of Highly Hydrophobic Plant Oil-Based Monomers with Sodium Dodecyl Sulfate

The synthesized POBMs contain CH_2_=CH–C(O)–NH–CH_2_CH_2_–O–C(O)– as a hydrophilic fragment in their chemical structure and R– as a hydrophobic constituent in the fatty acid chain. Such a monomer structure imparts unique properties to the POBMs associated with the ability to participate in intermolecular (electrostatic and/or van der Waals) interactions to produce complexes (aggregates).

**Figure 12:**
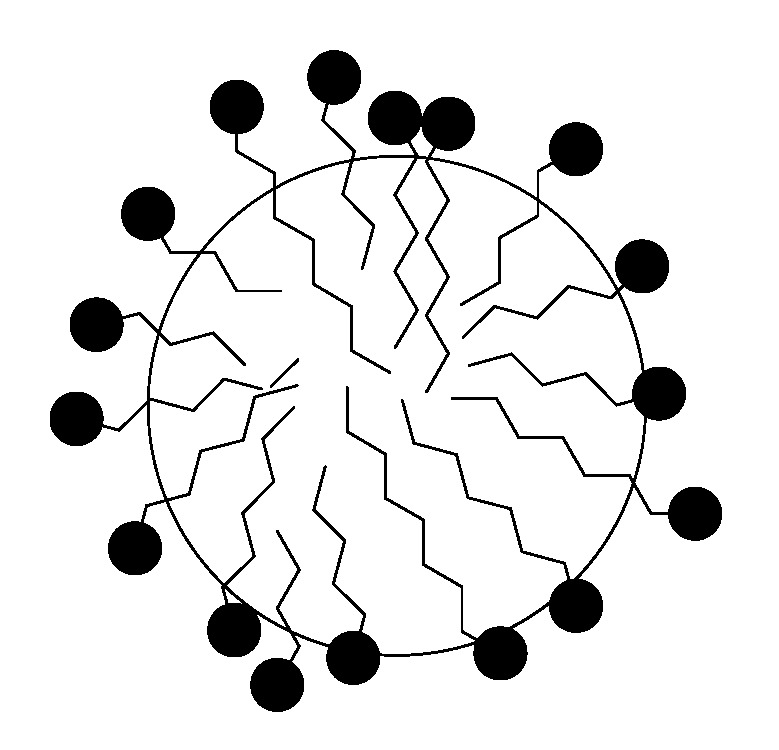
Possible location of the polar “heads” in a direct micelle from the SDS molecules and the POBM/SDS aggregate.

The influence of high oleic soybean oil-based (HO-SBM) and olive oil-based acrylic monomers on micellization of sodium dodecyl sulfate (SDS) was studied at different monomer and surfactant concentrations. The obtained data imply that SDS is able to solubilize highly hydrophobic monomer molecules. This leads to development of mixed (SDS/POBM) micelles (Figure 12). Surface activity of a SDS/POBM mixture varies by adding the monomer and, as a rule, is higher than for the surfactant alone [36]. It was also found that monomer molecules replace surfactant counterparts in the mixed micelles. Both the DLS and TEM data allow for conclusion that incorporation of POBM into the mixed micelles facilitates micellar association and development of 25–30 nm structures. Hence, solubilization of monomer molecules by SDS can affect mechanism of emulsion polymerization and reaction kinetics, as well as have an effect on development of latex particles and their morphology.

Surfactants form direct micelles due to the interactions between hydrocarbon parts of their molecules. Usually, the micelle has a layered structure depending on the packaging of surfactant molecules. A micelle contains a hydrocarbon nucleus, a water-hydrocarbon layer, which includes 2–3 methylene groups, and a layer of hydrated polar groups. It should be noted that surfactant molecules are freely localized in the micelle. They can leave the micelle or move to a different position; they can even be engulfed into the nucleus, which is determined by the hydrophobicity of the surfactant fragment [37,38].

**Figure 13:**
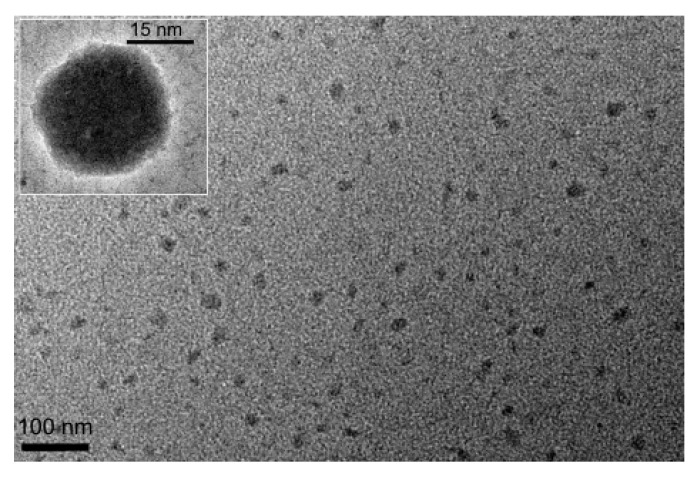
TEM micrograph of micelles prepared by mixing SDS (0.02M) and HO-SBM (0.01M) (inset shows morphology of selected individual micelle). Reprinted from Ref. 36, Copyright (2019), with permission from Elsevier.

The feature of micellization of sodium dodecyl sulfate (SDS) in the presence of the POBMs is surfactant ability to solubilize highly hydrophobic monomer molecules and promote development of mixed (SDS/POBM) micelles. As shown by Kingsley et al. [36], the packing of surface-active SDS/POBM complexes (aggregates) and the SDS molecules occurs upon micellization. In this event, the SDS molecules can be squeezed out of the micelle and localized at the interphase reducing the interfacial tension. Sodium dodecyl sulfate is known to have a micelle aggregation number of 60–70 [38]. It was found that the aggregation number decreases to 10–48 (Table 3) upon micellization of the SDS/POBM complexes. The micellar size depends on the packaging of the surfactants. Their length and packing density in the micelle determine the radius of the hydrocarbon nucleus. The formed colloidal solution simultaneously contains the SDS micelles and the mixed SDS/POBM micelles. It should be noted that their size is 18–38 nm (Table 3, Figure 13) whereas the SDS micellar size is 1.7–3.1 nm.

**Figure 14:**
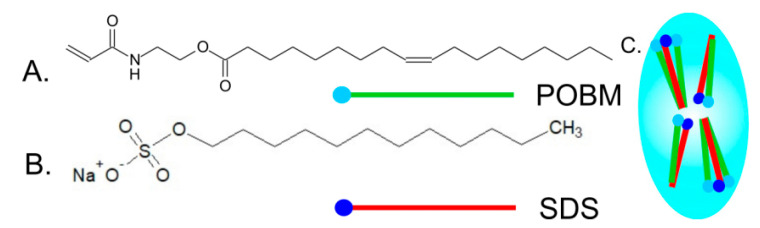
Chemical structure of plant oil-based monomer (**A**), surfactant (**B**), and schematic of POBM solubilization by SDS molecules (**C**). Reprinted from [36]. Copyright (2019), with permission from Elsevier.

If the fatty acid chain cannot leave the hydrocarbon nucleus, the polar group can be even drawn into the hydrophobic nucleus [38], which also affects the aggregation number. The authors explain the features of micellization (micellar parameters, size, structure, and surface tension) and the obtained results with the formation of surface-active SDS/POBM complexes (aggregates) of the following structure (Figure 14):

The formation of a water-hydrocarbon layer of the spherical micelle leads to the localization of the acrylic groups of the POBM molecule at the interface. This opens up new possibilities for the formation of “core–shell” morphology, providing ability of POBM to undergo polymerization.

Chemical structure of POBM and SDS molecules is similar. They both have hydrophilic “head” and long non-polar “tail” with in average 17 (POBM) and 12 (SDS) carbon atoms. It was suggested that physical association of both polar “heads” and hydrophobic “tails” in water underlie intermolecular interactions. The results indicate that surface activity of a SDS/POBM mixture is usually higher than for the surfactant and varies by adding the monomer. Moreover, structure, size, and micellar parameters, observed changes upon the addition of the POBMs. The formed complexes can open up new opportunities for micellization and, thus, new approaches to conducting heterophase polymerization (e.g., conventional emulsion and miniemulsion polymerization).

## 6. Dual Role of Methyl-β-Cyclodextrin in the Emulsion Polymerization of Highly Hydrophobic Plant Oil-Based Monomers

The new acrylic monomers are also capable of complexation with cyclodextrins, which play a dual role in both protecting against allylic termination chain transfer as well as improving monomer polymerizability.

Methyl-β-cyclodextrin (M-β-CD), amphiphilic oligosaccharide, was utilized to enhance the polymerizability of high oleic soybean oil-based and linseed oil-based monomers in copolymerization with styrene. Interactions between M-β-CD and the POBMs leading to the development of 1:1 “host-guest” complex were revealed. In the presence of the oligosaccharide, polymer yield rises whereas the coagulum content decreases during the emulsion polymerization of POBMs with styrene implying their improved solubility in water. Latex polymers with a higher molecular weight were synthesized in the presence of inclusion complexes. Incorporation of POBM molecules into the M-β-CD cavities protects the fatty acid chains and reduces chain transfer as confirmed by ^1^H-NMR spectroscopy. This effect is more pronounced for more unsaturated linseed oil-based monomer.

**Figure 15:**
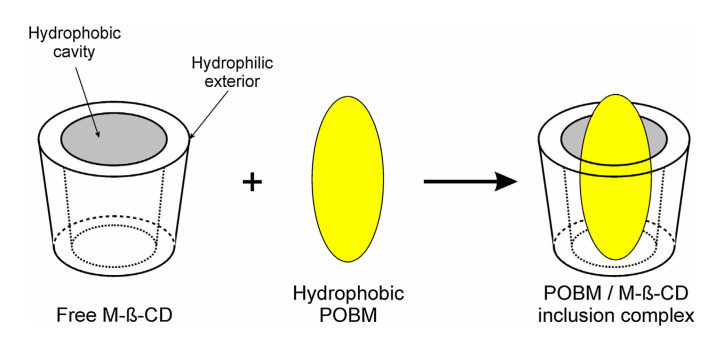
Schematic illustration of the association of free methyl-β-cyclodextrin and POBM to form a 1:1 inclusion complex. Reprinted from [32]. Copyright (2018), with permission from Elsevier.

These results are explained by the specific structures of M-β-CD having a hydrophobic cavity and a hydrophilic exterior, and the POBM molecule, possessing a long hydrophobic fatty acid chain; this results in the formation of an inclusion complex (Figure 15).

**Figure 16:**
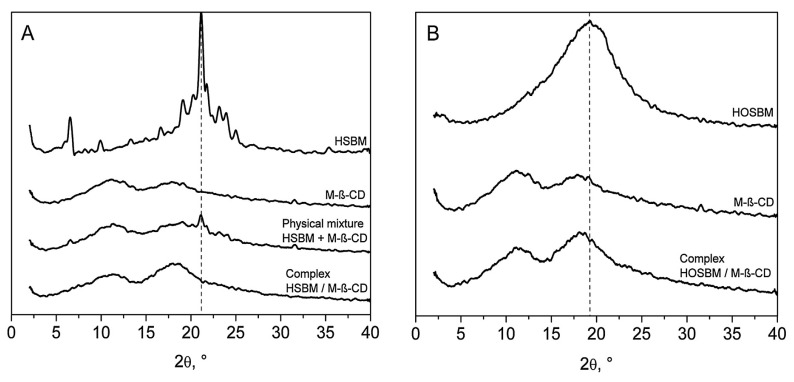
PXRD spectra of POBMs, methyl-β-cyclodextrin, their physical mixture, and inclusion complexes (**A**) – H-SBM, (**B**) – HO-SBM. Reprinted from [32]. Copyright (2018), with permission from Elsevier.

To confirm the formation of the inclusion complex, it was used Powder X-ray diffractometry (PXRD) [39]. The PXRD spectra of H-SBM [monomer from hydrogenated (very low degree of unsaturation) soybean oil], M-β-CD, H-SBM/M-β-CD inclusion complex, and the physical mixture are depicted in Figure 16A. The diffractogram of the physical mixture of M-β-CD and H-SBM was the superposition of the monomer and the oligosaccharide implying that there is no interaction between them [40]. The diffraction pattern of the H-SBM/M-β-CD inclusion complex displayed no characteristic peaks that the pure monomer had. These data reveal that the self-lattice arrangement of the monomer was changed from a crystalline to amorphous state due to the H-SBM inclusion into the methyl-β-cyclodextrin cavity.

The diffractograms of HO-SBM, M-β-CD, and HO-SBM/M-β-CD inclusion complex are shown in Figure 16B. As both M-β-CD and high oleic soybean oil-based monomer are non-crystalline substances, the development of HO-SBM/M-β-CD inclusion complex cannot be proven by PXRD.

**Figure 17:**
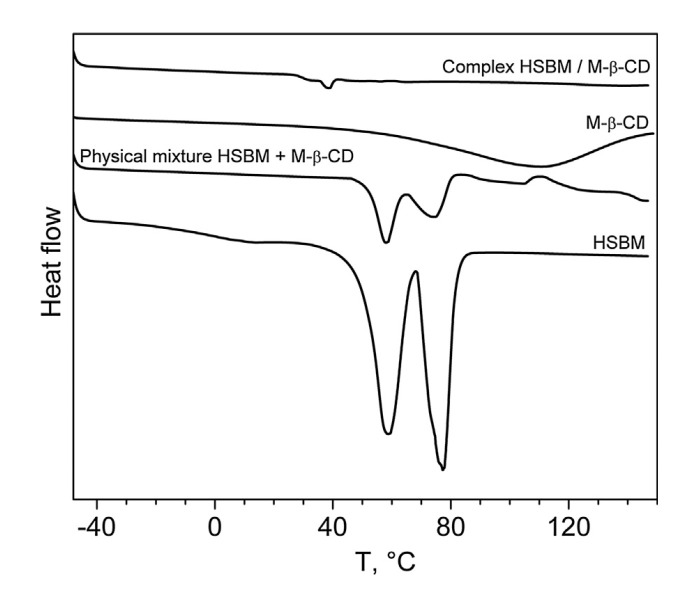
DSC thermograms of hydrogenated soybean monomer, methyl-β-cyclodextrin, their physical mixture, and inclusion complex. Reprinted from [32]. Copyright (2018), with permission from Elsevier.

To this end, differential scanning calorimetry [41] was used to further confirm the complexation. The DSC thermograms of H-SBM, M-β-CD, H-SBM/M-β-CD inclusion complex, and the physical mixture are shown in Figure 17. Although the endothermic peaks of the monomer were detected in the DSC curve of the physical mixture, no peak in the melting range of the monomer was detected in the thermogram of H-SBM/M-β-CD. This confirms that the H-SBM molecules are entirely included into the oligosaccharide cavities and H-SBM/M-β-CD inclusion complex forms.

No peak in the melting range of high oleic soybean oil-based monomer was observed in the DSC curves of HO-SBM/M-β-CD inclusion complex and their physical mixture (data not shown). Obviously, this is because the HO-SBM molecules might be able to penetrate into the M-β-CD cavities since the monomer is an oily liquid. This phenomenon is known as the kneading method for the formation of cyclodextrin-guest complexes [42].

Even though PXRD and DSC studies confirmed the transitions from a crystalline to amorphous state of H-SBM upon its interaction with M-β-CD, these analyses neither definitely confirmed inclusion complexation (especially, for HO-SBM) nor determined the complex stoichiometry. Thus, electrospray ionization–mass spectrometry (ESI–MS) [42,43] was used to investigate the complexation of M-β-CD with POBM.

**Figure 18:**
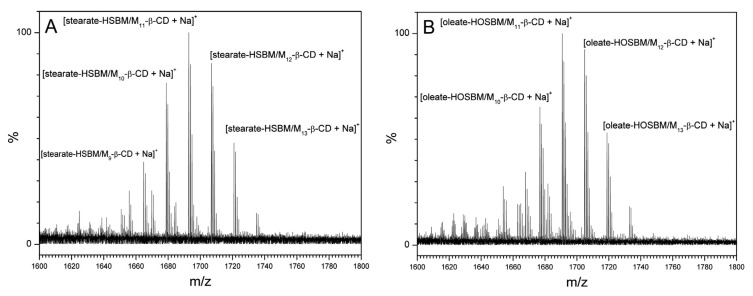
ESI mass spectra of H-SBM/M-β-CD (**A**) and HO-SBM/M-β-CD (**B**) inclusion complexes. Reprinted from [32]. Copyright (2018), with permission from Elsevier.

The ESI mass spectra of inclusion complexes of the monomers with M-β-CD are presented in Figure 18. Ions detected in the spectrum of H-SBM/M-β-CD (Figure 18A) are attributed to sodium adducts of complexes of stearate-H-SBM with M-β-CD having 9–13 methyl groups. In the ESI mass spectrum of the HO-SBM/M–β–CD inclusion complex (Figure 18B), ions that correspond to the inclusion of oleate-HO-SBM by M_10-13_-β-CD (sodium adducts) are revealed.

Thus, the M-β-CD complexation of monomer molecules leading to the 1:1 complex formation (Figure 15) was directly confirmed by the ESI–MS data. The emulsion copolymerization of the POBMs with styrene in the presence of methyl-β-cyclodextrin was found to allow for the preservation and protection of fatty acid double bonds (–CH=CH–) and the bisallylic hydrogen atoms (–CH=CH–CH_2_–CH=CH–). The inclusion complexation decreases the chain transfer contribution by protecting the allylic moiety of the monomer fatty acid chains and simultaneously improves the aqueous solubility and availability of the monomers in the emulsion polymerization process. Therefore, a higher molecular weight of latex polymers from the highly hydrophobic POBMs was achieved with higher monomer conversion and lower coagulum formation.

## 7. Conclusions

Monomers based on various plant oils were synthesized through a two-step procedure. A one-step process for the synthesis of new vinyl monomers by the reaction of direct transesterification of plant oil triglycerides with *N*-(hydroxyethyl)acrylamide was recently patented. The features of homopolymerization kinetics for the POBMs were determined. The reactivity of the plant oil-based monomers was studied by radical copolymerization with vinyl monomers. The Alfrey−Price Q (0.41−0.51) and e (0.09−0.28) parameters are close for all POBMs and do not essentially depend on the monomer structure. The unique molecular structure of the plant oil-based acrylic monomers is that they simultaneously have a CH_2_=CH–C(O)–NH–CH_2_CH_2_–O–C(O)– polar (hydrophilic) fragment capable of electrostatic interactions and a hydrophobic fatty acid chain (C15–C17) capable of van der Waals interactions. This opens up new opportunities for formation of micelles from the complexes of highly hydrophobic plant oil-based monomers with sodium dodecyl sulfate and new approaches to conducting heterophase polymerization.

Preparation of polymeric coatings from the plant oil-based monomers through the incorporation of the hydrophobic fatty acid chains into the macromolecules of latex polymers allows for formation of polymer networks with a controlled cross-linking density along with enhancing the water resistance of the coatings. The copolymers based on the olive and soybean monomers enable the formation of coatings with low surface energy and water-repellent properties. The copolymers based on the olive and soybean monomers enable the formation of coatings with low surface energy and water-repellent properties.

The formation of inclusion complexes upon the interaction between the molecules of the plant oil-based acrylic monomers and methyl-β-cyclodextrin leads to a higher molecular weight of latex polymers from highly hydrophobic POBMs with higher monomer conversion and lower coagulum formation.

An analysis of the literature shows a rapid development of a new branch in polymer science related to the chemistry of aliphatic and aromatic biobased monomers from renewable plant sources. The synthesis of such monomers allows for producing fully biobased polymers with biocompatible and biodegradable properties which do not pollute the environment. Copolymerization of the biobased monomers with commercial monomers enables formation of copolymers with controlled physical, chemical, and mechanical properties.

## 8. Patents

Biobased Acrylic Monomers US 10,315,985 B2 June 11th, 2019.

Biobased Acrylic Monomers and Polymers Thereof US 10,584,094 B2 March 10th, 2020.

## Figures and Tables

**Table 1:** Content of fatty acid chains in olive oil.

Fatty Acid Chains	Signal (Protons)	Field Correction Factor		Content of Fatty Acids in the Oil, %	
			%	Calculated	Literature data
Linolenate (E)	0.95–1.05 ppm (-CH_3_)	2H/9H	22.2	0.73	<1%
Linoleate (A)	2.75–2.85 ppm (-CH=CH-CH_2__-_CH=CH-)	2H/6H	33.3	8.06 − 2∙0.73 = 6.6	3.5–21
Oleate (C)	1.97-2.11 ppm (-CH_2_-CH=CH-CH_2_-)	2H/12H	16.7	87.56 − (0.73 + 6.6) = 80.23	55–83%
Content of saturated fatty acid chains:				100 − (0.73 + 6.6 + 80.23) = 12.44	1–20%

**Table 2:** Physico-chemical characteristics of POBMs.

Monomer from Oil	Content of Unsaturated Fatty Acids, %			Iodine Value (Same for Oil), g/g	Molar Mass, g/mol
	C_18:1_	C_18:2_	C_18:3_		
High Oleic Sunflower Oil (HOSFM)	86–89	3–6	0.5–1	105 (82)	379.0 ^1^
Olive Oil (OVM)	65–85	3.5–20	0–1.5	110 (90)	379.3 ^2^
High Oleic Soybean Oil (HOSBM)	70–73	13–16	0–1	124(105)	379.3 ^2^
Canola Oil (CLM)	60–63	18–20	8–10	137 (96)	379.0 ^2^
Corn Oil (COBM)	23–31	49–62	0–2	139 (120)	377.0 ^1^
Sunflower Oil (SFM)	14–35	44–75	0–1	146 (128)	377.5 ^2^
Soybean Oil (SBM)	22–34	43–56	7–10	149 (139)	377.3 ^2^
Linseed Oil (LSM)	12–34	17–24	35–60	194 (177)	375.6 ^1^

^1^ Calculated. ^2^ Experimental.

**Table 3:** Micellar parameters for SDS and POBM at different concentrations.

SDS + POBM (x,mol: y,mol)	*N_agg_*	*N_POBM_*	*I_1_/I_3_*	d,nm	PDI	σ, mN/m
SDS at 0.02 M	41	0	1.04	3.1	0.006	36.2
+ HO-SBM 0.01 M	25	20	0.94	18.2	0.02	34.1
0.02 M	15	25	0.94	23.3	0.05	31.2
0.04 M	12	39	0.92	25.4	0.04	30.4
SDS at 0.05 M	57	0	1.03	1.7	0.003	34.9
+ HO-SBM 0.02 M	46	22	0.96	27.3	0.03	31.5
0.04 M	38	34	0.95	28.5	0.05	29.8
SDS at 0.02 M	41	0	1.03	3.1	0.006	36.2
+ OVM 0.01 M	27	22	0.95	22.6	0.06	32.4
0.02 M	19	31	0.93	28.7	0.04	31.1
0.04 M	10	33	0.93	37.8	0.04	30.1
SDS at 0.05 M	57	0	1.03	1.7	0.003	34.9
+ OVM 0.02 M	48	23	0.95	28.2	0.02	32.6
0.04 M	31	28	0.94	33.9	0.04	30.2
